# Psychosocial Factors That Influence the Health of Workers in Contemporary Workplaces

**DOI:** 10.3390/ijerph192114016

**Published:** 2022-10-27

**Authors:** Silvia Pignata

**Affiliations:** Centre for Workplace Excellence & STEM Unit, University of South Australia, Mawson Lakes, SA 5095, Australia; silvia.pignata@unisa.edu.au

## 1. Introduction

This Special Issue of the *IJERPH* examines various psychosocial factors that influence the health of workers in contemporary workplaces. The American Psychological Association [1] defines psychosocial as “the intersection and interaction of social, cultural, and environmental influences on the mind and behavior”. Clearly, the onset of COVID-19 in 2020 and the resultant changes in how people live and work are strong examples of psychosocial influences. The impact of and speed at which remote work was initiated to enable people to work from home provided challenges in not only the necessary support, infrastructure, and skills required to do so, but also the associated communication and coordination costs of enabling workers to work effectively [2] and for managers to successfully manage teams as well as maintain productivity. There is a growing realization in the corporate world, as well as with regulators in developed countries, that management interest in and commitment to occupational health and safety benefits both workers and organizational productivity [3]. As a result, mental health and psychosocial work environments are now on the corporate agenda and deserve further research.

## 2. Discussion

When examining psychosocial factors in the workplace, it is important to take a holistic approach that encompasses both individual and organizational components. Key factors in mentally healthy workplaces include the ability to have good relationships between management and workers [4]; effective leadership [5,6]; support and trust in management and institutions [7]; a strong organizational culture (including safety culture) [8]; family support and reduced work–family interference [9]; and recognition of the changing world of work itself [10].

The Special Issue attracted papers from researchers around the world. After reviewing all of the submissions, 16 papers met the criteria for blind peer review, and, of these, 13 papers were published, representing research from China, Germany, Italy, Malaysia, South Korea, Spain, Taiwan, the United Kingdom (UK), and the United States (US). This editorial provides an overview of these papers, which include longitudinal studies on (1) the prediction of depressive symptoms; (2) family support during COVID-19; and (3) the relationship between the family-to-work interface and workplace injuries. Other topics are telecommuting, leadership and well-being, role conflict, patient safety culture and safety performance, gender and gender role orientation in demands and work–family interference, the potential of job demands during change, institutional trust during COVID-19, sickness presenteeism in prison officers, a new tool to measure academics’ quality of life, and remote work in a changing world.

In her commentary, *“Remote Work in a Changing World: A Nod to Personal Space, Self-Regulation and Other Health and Wellness Strategies”,* Geldart suggests how to make remote work more satisfying, safe, and healthy for workers. She asserts that workers and management need to work together to enhance at-home occupational health and safety, so that (1) workers have a personal work area with adequate space, lighting, and ventilation; (2) management considers ergonomics and provides assessments as well as equipment for home offices; and (3) workers are able to self-regulate and learn skills by using new technologies, setting work goals, and staying on task.

The effect of telecommuting on workers’ health is examined by Magnavita and colleagues in *“Telecommuting, Off-Time Work, and Intrusive Leadership in Workers’ Well-Being”.* They surveyed 905 Italian workers from companies that had limited telecommuting to < 10 h per week to investigate intrusive leadership. They found that intrusive leadership and working after hours were significantly associated with occupational stress, and that intrusive leadership and overtime work were associated with reduced happiness, anxiety, and depression.

Leadership is further examined by Díaz-Fúnez and colleagues in their submission *“Are Job Demands Necessary in the Influence of a Transformational Leader? The Moderating Effect of Role Conflict”.* In a study on 705 workers in a Spanish multinational private company, they found a moderating effect of role conflict between the intellectual stimulation of employees and intellectual engagement in addition to a mediating effect of intellectual engagement between leadership behavior and employee performance. The researchers assert that organizations must offer an environment that challenges employees.

Chen and colleagues examined *“Post-Pandemic Patient Safety Culture: A Case from a Large Metropolitan Hospital Group in Taiwan”.* They report on a sample of 337 employees who participated in quality improvement interventions and then completed a questionnaire on patient safety culture and personal well-being. Their analyses showed that managerial role, seniority, female gender, and direct contact with a patient were significantly related to a positive attitude of safety culture. In the post-pandemic era, there was an improvement in patient safety culture among hospital staff, particularly for managers.

In another submission on safety culture, *“Impact of Safety Culture on Safety Performance; Mediating Role of Psychosocial Hazard: An Integrated Modelling Approach”,* Naji and colleagues surveyed 380 production employees from the oil and gas sector in three Malaysian states. Using structural equation modeling, they found that psychosocial hazards fully mediate the relationship between safety culture and safety performance, and that psychosocial concerns in workplace environments need to be considered by employees to enhance safety performance.

In the paper *“The Demand–Control Model as a Predictor of Depressive Symptoms—Interaction and Differential Subscale Effects: Prospective Analyses of 2212 German Employees”*, Burr and colleagues tested the demand–control model to investigate (a) the combination of low job control and high psychological demands on depressive symptoms in addition to (b) whether subscales of psychological demands and job control had similar associations with depressive symptoms. Analyses of a 5-year cohort of 2212 German workers did not show an interaction effect of low job control and high psychological demands on depressive symptoms. However, when the subscales were based on value ranges, they found differences in the job control subscales of decision authority and skill discretion.

Gender and gender role orientation (egalitarian vs. traditional) is examined by Lu and colleagues in the paper *“Do Gender and Gender Role Orientation Make a Difference in the Link between Role Demands and Family Interference with Work for Taiwanese Workers?”*. Responses from 251 Taiwanese employees revealed a stronger relationship between work demands and family-to-work conflict (FWC) for egalitarian men than traditional men. For women, the relationship between family demands and FWC was stronger for egalitarian women. The results highlight the uncertain position that egalitarian men and women in Chinese society have in fulfilling their work and family roles.

*“The Power of Family Support: The Long-Term Effect of Pre-COVID-19 Family Support on Mid-COVID-19 Work Outcomes”* was examined by Shin and colleagues. Their study proposes that family support provided before the onset of COVID-19 has a positive indirect effect on job performance and organizational citizenship behavior after its onset, by decreasing emotional exhaustion. They collected two-wave data from 211 South Korean employees over a 17-month period and found that pre-COVID-19 family support has a positive longitudinal effect on work outcomes for employees during the pandemic.

Siu and Ng examined *“Family-to-Work Interface and Workplace Injuries: The Mediating Roles of Burnout, Work Engagement, and Safety Violations”* by applying the job demands–resources and conservation of resources theories to evaluate the mediating roles of burnout, work engagement, and safety violations in the relationships of family-to-work conflict (FWC) and family-to-work enrichment (FWE) with workplace injuries. Their analysis of two-wave data from 233 Chinese nursing and rail employees shows that the relationship between FWE with workplace injuries was mediated by work engagement and safety violations. Burnout and safety violations were found to mediate the relationship between FWC with workplace injuries.

Research by Blum and Rigotti, titled *“When and Why Demands Reveal Their Challenging Potential during Change”*, examines how trust and autonomy can alter the dynamics of demands during change processes. They integrate the challenge–hindrance framework with the job demands–resources framework in a study on 388 employees experiencing organizational change. They find that both trust and autonomy can support how an individual assesses the extent to which the change impacts their job demands, expectations, and responsibilities. The findings assert the need to consider both organizational and personal factors when implementing change.

In their paper, *“In Whom Do We Trust? A Multifoci Person-Centered Perspective on Institutional Trust during COVID-19*”, Jiang and colleagues adopt a person-centered approach to examine institutional trust, with a focus on trust in the two different institutions of state and federal government. Using data from 492 US workers, they identified five trust profiles: high trustors, federal trustors, state trustors, the ambivalent, and distrusters. The authors show that these five profiles predicted attitudes and compliance with COVID-19 prevention practices, employee job insecurity, affective commitment, helping behavior, and psychological well-being.

In *“Sickness Presenteeism in Prison Officers: Risk Factors and Implications for Wellbeing and Productivity”*, Kinman and Clements report on the prevalence and occupational-, organizational-, and individual-level factors that encourage UK prison officers to work while sick. By analyzing survey data from 1956 prison officers, 92% reported working while unwell at least sometimes, with 43% reporting that they always did. As presenteeism can have severe safety implications for officers and prison inmates, these findings can inform the development of interventions to improve the culture of sickness absence in prisons.

Brondino and colleagues present *“A New Academic Quality at Work Tool (AQ@workT) to Assess the Quality of Life at Work in the Italian Academic Context”*. The tool is based on the job demands–resources theoretical framework and was developed by Italian academics who are also work and organizational psychologists. Three studies in academia demonstrate that the AQ@workT is a reliable and useful tool that can by employed by university management to mitigate work stress and enhance the quality of an academic’s personal and professional lives.

## 3. Conclusions

The breadth of these 13 articles provides an interesting overview of psychosocial issues in workplaces that encourage more research in this important area.
